# Varying gestational age patterns in cesarean delivery: an international comparison

**DOI:** 10.1186/1471-2393-14-321

**Published:** 2014-09-13

**Authors:** Marie Delnord, Béatrice Blondel, Nicolas Drewniak, Kari Klungsøyr, Francisco Bolumar, Ashna Mohangoo, Mika Gissler, Katarzyna Szamotulska, Nicholas Lack, Jan Nijhuis, Petr Velebil, Luule Sakkeus, James Chalmers, Jennifer Zeitlin, Euro-Peristat Preterm Group

**Affiliations:** INSERM UMR1153, Obstetrical, Perinatal and Pediatric Epidemiology Research Team, Research Center for Epidemiology and Biostatistics Sorbonne Paris Cité (CRESS), DHU Risks in pregnancy, Port Royal Maternity Unit, Paris Descartes University, 53 Avenue de l’Observatoire, Paris, 75014 France; Department of Global Health and Primary Health Care, University of Bergen, Bergen, Norway; Medical Birth Registry of Norway, Norwegian Institute of Public Health, Bergen, Norway; Department of Public Health Sciences, University of Alcalá, Madrid, Spain; Department Child Health, TNO Netherlands Organization for Applied Scientific Research, Leiden, The Netherlands; Department of Information, THL National Institute for Health and Welfare, Helsinki, Finland; NHV Nordic School of Public Health, Gothenburg, Sweden; Department of Epidemiology, National Research Institute of Mother and Child, Warsaw, Poland; Department of Methods and Perinatology, BAQ, Bavarian Institute for Quality Assurance, Munich, Germany; Department of Obstetrics and Gynaecology, MUMC, Grow school for oncology and developmental biology, Maastricht, The Netherlands; Institute for the Care of Mother and Child, Prague, Czech Republic; Estonian Institute for Population Studies, Tallinn University, Tallinn, Estonia; Information Services Division, NHS National Services Scotland, Edinburgh, UK

**Keywords:** Cesarean delivery (CD), Cross-national comparisons, Gestational age, Plurality, Mode of delivery, Euro-Peristat

## Abstract

**Background:**

While international variations in overall cesarean delivery rates are well documented, less information is available for clinical sub-groups. Cesarean data presented by subgroups can be used to evaluate uptake of cesarean reduction policies or to monitor delivery practices for high and low risk pregnancies based on new scientific evidence. We studied differences and patterns in cesarean delivery rates by multiplicity and gestational age in Europe and the United States.

**Methods:**

This study used routine aggregate data from 17 European countries and the United States on the number of singleton and multiple live births with cesarean versus vaginal delivery by week of gestation in 2008. Overall and gestation-specific cesarean delivery rates were analyzed. We computed rate differences to compare mode of delivery (cesarean vs vaginal birth) between selected gestational age groups and studied associations between rates in these subgroups namely: very preterm (26–31 weeks GA), moderate preterm (32–36 weeks GA), near term (37–38 weeks GA), term (39–41 weeks GA) and post-term (42+ weeks GA) births, using Spearman’s rank tests.

**Results:**

High variations in cesarean rates for singletons and multiples were observed everywhere. Rates for singletons varied from 15% in The Netherlands and Slovenia, to over 30% in the US and Germany. In singletons, rates were highest for very preterm births and declined to a nadir at 40 weeks of gestation, ranging from 8.0% in Sweden and Norway, to 22.5% in the US. These patterns differed across countries; the average rate difference between very preterm and term births was 43 percentage points, but ranged from 14% to 61%. High variations in rate differences were also observed for near term versus term births. For multiples, rates declined by gestational age in some countries, whereas in others rates were similar across all weeks of gestation. Countries’ overall cesarean rates were highly correlated with gestation-specific subgroup rates, except for very preterm births.

**Conclusions:**

Gestational age patterns in cesarean delivery were heterogeneous across countries; these differences highlight areas where consensus on best practices is lacking and could be used in developing strategies to reduce cesareans.

## Background

Over the past decades, the percentage of cesarean deliveries has risen in almost all high-income countries, and the most recent European Perinatal Health Report with 2010 data confirms continuing increases [1, 2]. Researchers and clinicians have expressed concerns over these increasing rates and the impact on mothers’ and newborns’ health [2–4]. While cesarean delivery can be a lifesaving intervention for both mother and child, [5] it has been associated with significant neonatal and maternal adverse health outcomes in comparison with vaginal delivery [3, 4, 6–9]. Studies have also linked cesarean delivery with complications in subsequent pregnancies for women (placentation disorders, uterine rupture and stillbirth) [7, 8] as well as adverse long term child health outcomes such as asthma and type-1 diabetes [10, 11]. Given these risks and concerns about the optimal use of health resources, professional societies in many high income countries recommend strategies to reduce unnecessary cesareans [4, 12].

International comparisons of cesarean rates highlight differences in delivery practices and provide useful benchmarks for the evaluation of national practices. Such comparisons have been made using data in international databases run by Eurostat, OECD and WHO but these organizations collect overall cesarean rate data whereas comparisons stratified by risk groups may better inform cesarean reduction policies [5, 13, 14]. The Robson Ten-Group classification is a robust system which allows comparisons of cesarean rates based on characteristics of pregnancies, [15] but the data needed to identify cesareans based on this system are not available in any of the international databases and may be difficult to obtain in some countries on the national level.

Presenting rates by gestational age distinguishes between practices for higher risk (very preterm singletons and multiples) and lower risk infants (singletons at 39 and 40 weeks of gestation) and could be used by clinicians and health planners to evaluate strategies as well as uptake of cesarean reduction policies targeting specific sub-groups – for instance, near term pregnancies. We thus compared singleton and multiple cesarean rates across the gestational age continuum using data from the European countries participating in the Euro-Peristat project and the United States to investigate cross-national differences in delivery practices for very preterm, moderate preterm, near term, term and post term pregnancies in 2008.

## Methods

The Euro-Peristat project collects population-based aggregate data from European countries, on a set of core and recommended perinatal health indicators and also conducts *ad hoc* studies based on supplementary detailed data [16]. Scientific committee members (clinicians, statisticians, or researchers) from each participating country are responsible for data collection. The European data used in this analysis are from a study on preterm birth [16]. We requested data on the number of live births at or after 22 weeks for each completed week of gestation by multiplicity and mode of delivery for several years of which the most recent was 2008. We requested gestational age data based on the best obstetric estimate.

The US data used in this analysis were downloaded from the Center for Disease Control website (http://www.cdc.gov/nchs/data_access/Vitalstatsonline.htm). We used birth certificate data for live births in 2008 and extracted the variables on gestational age, multiplicity and mode of delivery. The clinical estimate of gestational age has been shown to include fewer birth weight extremes [17] and has been used successfully in other cross-country studies [18].

Seventeen European countries participated in the study and provided data on births in 2008, or the closest available time point. Data from France and Spain are from 2010; in Malta and Sweden data were provided for 2009. Three countries provided data on selected regions only: Germany provided data from three Länder, Belgium for Flanders, and the United Kingdom for Scotland only. Births with unknown mode of delivery were excluded from the analyses; births for which gestational age was missing were included in the computation of overall cesarean rates only. In total, we included 4,092,381 live singleton births and 143,777 live multiple births from the United States and 1,501,575 live singleton births and 55,550 live multiple births from Europe. For less than 0.5% of cases, gestational age data were missing. Our analysis was done on live births to improve the comparability of our estimates; stillbirths were excluded from the study due to differences in registration criteria across countries [18], and the distinct obstetric management of stillbirths versus live births [19].

For the analyses, we computed countries’ overall cesarean rates per 100 live births as well as rates for singleton and multiple births; we further calculated rates per completed week of gestation starting at 26 weeks [19, 20] in order to reduce variability resulting from small sample sizes and differences in management at the earlier gestational ages. We did not display data with less than five cesareans in a given cell. For singletons, we presented rates for each individual week of gestation and for very preterm births: 26+0 - 31+6 weeks GA, moderate preterm births: 32+0 - 36+6 weeks GA, near-term: 37+0 - 38+6 weeks, term: 39+0 - 41+6 weeks and post-term births: 42+0 weeks GA and over. For multiples, we used different gestational age cut-offs to account for the increased risk of premature delivery and for the small number of births at the later gestational ages. Due to small sample sizes for twins in our participating countries, we only presented rates at grouped weeks of gestational age: below 34 weeks GA, 34+0 - 36+6 weeks, and 37+0 weeks and over [20].

Next, we computed rate differences in each country to compare cesarean practices for term pregnancies to those for near term and very preterm pregnancies. We accounted for variations in the number of births by presenting rate differences with their 95% confidence intervals. We calculated the mean cesarean rate difference between very preterm and term singleton births and between near term and term singleton births. We also computed the mean cesarean rate difference between multiple births before 34 weeks and after 36 weeks of gestation. Last, we examined the associations between cesarean rates in singletons and multiples and the overall cesarean rate using Spearman’s rank tests; we carried out this analysis in each of our gestational age subgroups. Data were analyzed using STATA 10.0 software (StataCorp LP, College Station, TX, USA).

## Results

Overall cesarean rates ranged from 15.7% to 32.5% of all live births (Table 1). For singleton births, Germany and the United States had the highest rates, 31.0% and 30.8% respectively, while Slovenia and The Netherlands had the lowest rates (15.5% and 14.8% respectively). For multiples, Austria and Malta had the highest rates, 83.4% and 95.4% respectively while The Netherlands and Norway had the lowest rates, 39.6% and 46.7% respectively.

**Table 1:** **Cesarean delivery rates by plurality in 2008**

Country/Region	Overall			Singletons			Multiples		
	Live births (N)	Rate %	95% CI	Live births (N)	Rate %	95% CI	Live births (N)	Rate %	95% CI
Austria	77 728	28.0	27.7-28.3	75 072	26.0	25.7-26.4	2656	83.4	81.9-84.8
Belgium: Flanders	69 187	20.2	20.0-20.5	66 672	18.8	18.5-19.1	2515	56.7	54.7-58.6
Czech Republic	119 455	22.0	21.7-22.2	114 722	19.7	19.5-20.0	4733	75.4	74.1-76.6
Estonia	16 032	21.2	20.5-21.8	15507	19.3	18.7-19.9	525	77.5	73.7-81.0
Finland	59 594	17.2	16.9-17.5	57 872	16.2	15.9-16.5	1722	51.9	49.5-54.2
France^1^	14 594	21.1	20.4-21.7	14 161	20.1	19.4-20.7	433	54.3	49.4-59.0
Germany^2^	211 889	32.5	32.3-32.7	204 746	31.0	30.8-31.2	7143	74.6	73.6-75.6
Ireland	75 249	26.7	26.3-27.0	72 592	25.3	25.0-25.6	2657	64.0	62.1-65.8
Lithuania	31 287	23.9	23.4-24.4	30 510	22.9	22.4-23.3	777	63.3	59.8-66.7
Malta^3^	4152	30.2	28.8-31.6	4020	28.1	26.7-29.5	132	95.4	90.4-98.3
The Netherlands	174 828	15.7	15.5-15.6	168 619	14.8	14.6-15.0	6209	39.6	38.4-40.8
Norway	61 206	17.2	17.0-17.5	59 075	16.2	15.9-16.5	2131	46.7	44.6-48.9
Slovakia	53 971	25.7	25.3-26.0	52 520	24.2	23.9-24.6	1451	77.5	75.2-79.6
Slovenia	21 806	16.9	16.4-17.4	21 041	15.5	15.0-16.0	765	54.9	51.3-58.5
Spain^4^	398 922	27.4	27.3-27.5	382 141	25.5	25.3-25.6	16 781	71.3	70.6-72.0
Sweden^3^	108 923	18.0	17.8-18.3	105 855	17.0	16.7-17.2	3068	54.6	52.8-56.3
UK: Scotland	58 302	26.1	25.8-26.5	56 450	24.9	24.5-25.2	1852	64.7	62.5-66.9
USA	4236158	32.4	32.3-32.4	409 2381	30.8	30.8-30.9	143777	76.4	76.2-76.6

^1^Data from France came from a nationally representative survey of all births in 2010.

^2^Data from Germany are from 3 Landers.

^3^Data from Malta and Sweden are from 2009.

^4^Data from Spain are from 2010.

**Figure 1:**
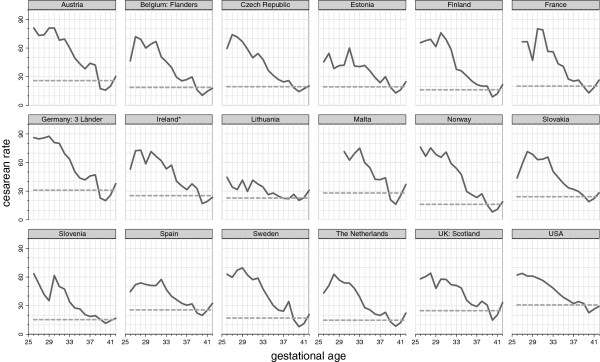
**Cesarean rates for singleton births overall and by gestational age at delivery in 2008.** Legend: ---------------- overall cesarean rate  cesarean rate by GA in completed weeks.

**Figure 2:**
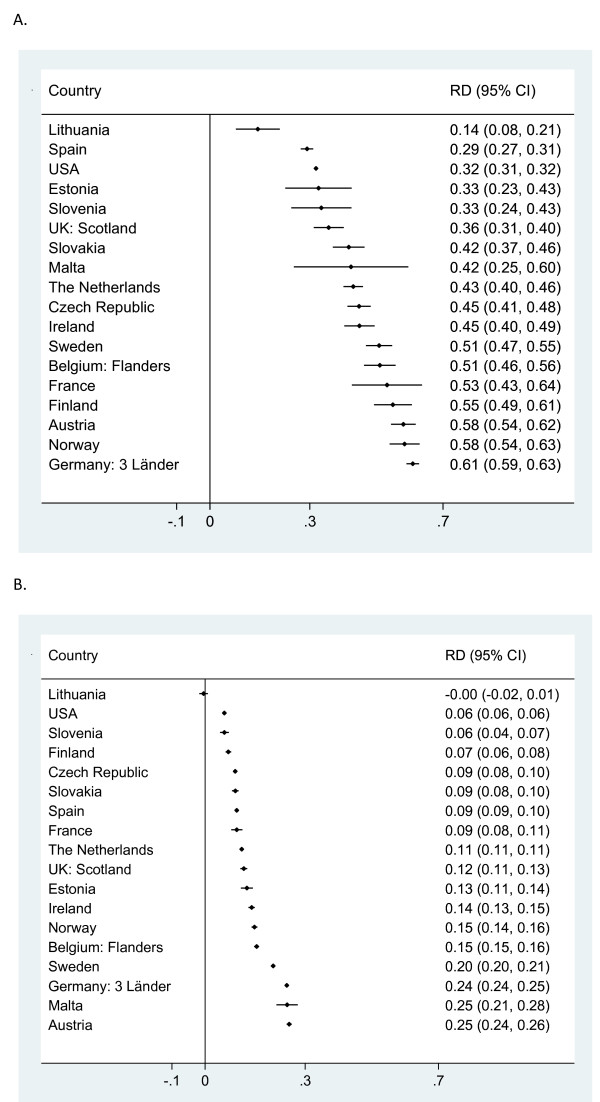
**Cesarean rate differences by gestational age groups for singletons in 2008. A**. Rate differences between very preterm (26–31 weeks GA) and term (39–41 weeks GA). **B**. Rate differences between near term (37–38 weeks GA) and term (39–41 weeks of GA).

In Figure 1 we display cesarean rates by week of gestation for singleton births; the dotted line shows the overall rate. Rates were highest for very preterm deliveries ranging from 37.0% in Lithuania to 83.3% in Germany (Table 2). Rates decreased with increasing gestational age to a nadir at 40 weeks of gestation, ranging from lows of 8.0% in Sweden and 8.4% in Norway to a high of 22.5% in the US, before rising at 41 and 42 weeks. In a majority of countries, there was also an increase in cesarean deliveries at 38 weeks; this rise was pronounced in Ireland, Sweden, and Estonia. Rates declined everywhere with increasing gestational age but depending on the country, rate differences between preterm, near term and term births differed.The rate differences presented in Figure 2 measure the range in delivery practices by gestational age across countries for singletons which were observed in Figure 1. We compared rates for two groups at higher risk of cesarean, namely very preterm and near term births, with rates in term births. The average rate difference between very preterm and term births was 43 percentage points, with a range from 14.4% in Lithuania to 61% in Germany as illustrated in Figure 2A. Figure 2B shows that a great heterogeneity also existed between near term and term singleton births; while the average rate difference was 12 percentage points, it ranged from 0.4% in Lithuania to 25.2% in Austria. Some of the variability in early gestational ages also reflects random variation among countries with smaller number of births.

**Table 2:** **Cesarean delivery rates by plurality and gestational age subgroups in 2008**

Singletons						Multiples		
Gestational age in completed weeks (wks)								
	26-31 wks	32-36 wks	37-38 wks	39-41 wks	≥42 wks	<34 wks	34-36 wks	≥37 wks
Country/Region	%	%	%	%	%	%	%	%
Austria	75.5	44.7	42.6	17.4	30.3	89.8	84.5	76.0
Belgium: Flanders	64.6	31.2	29.0	13.6	18.1	61.0	57.5	53.9
Czech Republic	61.3	32.7	25.6	16.5	20.4	82.3	75.9	71.6
Estonia	48.4	34.7	28.4	15.8	24.9	66.3	77.8	81.7
Finland	68.6	31.2	20.6	13.7	21.9	67.4	48.5	48.2
France^1^	69.9	36.5	26.2	16.7	26.5	56.4	56.3	52.8
Germany^2^	83.3	46.4	46.8	22.3	37.9	87.8	76.1	64.8
Ireland	67.0	43.1	36.0	22.1	23.6	67.4	68.2	60.1
Lithuania	37.0	27.4	22.2	22.6	31.1	71.2	66.0	59.0
Malta^3^	61.3	52.0	43.5	18.9	37.0	85.7	92.3	100
The Netherlands	54.2	26.3	22.2	11.2	22.4	40.8	39.4	39.3
Norway	70.0	34.6	26.3	11.5	18.9	59.0	47.5	42.3
Slovakia	62.9	39.4	30.3	21.2	28.2	76.4	79.3	76.6
Slovenia	47.0	25.9	19.3	13.5	16.7	56.0	58.0	50.9
Spain^4^	51.2	36.7	31.5	22.1	32.5	73.8	71.7	70.0
Sweden^3^	62.1	32.0	31.7	11.3	21.0	71.0	56.0	48.9
UK: Scotland	57.1	37.9	33.0	21.4	33.3	64.7	69.5	61.4
USA	59.8	39.2	33.7	28.0	29.3	81.0	76.8	73.1

^1^Data from France came from a nationally representative survey of all births in 2010.

^2^Data from Germany are from 3 Landers.

^3^Data from Malta and Sweden are from 2009.

^4^Data from Spain are from 2010.

Data on multiple births in Table 2 show rates of cesarean section by grouped weeks of gestation. In Finland, Germany, Norway, and Sweden, rates declined by gestational age whereas in others: Slovakia, UK, and The Netherlands, rates were more similar across all weeks of gestation. The average rate difference between babies born before 34 weeks and those born after 37 weeks was 7 percentage points and ranged from 0.2% in Slovakia to 23.0% in Germany.

Table 3 displays the correlation between cesarean rates in singletons and multiples and the overall rate; we examined these associations in each of our gestational age categories. Across countries, the overall cesarean rate was very highly correlated with all other subgroup rates (r = 0.8, p = 0.0), with the exception of very preterm singleton deliveries (r = 0.16, p = 0.52). Singleton and multiple subgroup rates were moderately to highly correlated with each other. Notably in singleton births, rates in moderate preterm births were most correlated with rates in near term births, and rates in post-term pregnancies were most correlated with rates in term pregnancies. Cesarean rates for very preterm singletons were not correlated with any other subgroup rates.

**Table 3:** **Spearman correlation coefficients for cesarean rates by plurality and gestational age subgroups in 2008**

Subgroups	Overall	Singletons					Multiples		
	≥22 wks	26-31 wks	32-36 wks	37-38 wks	39-41 wks	≥42 wks	<34 wks	34-36 wks	≥37 wks
Singletons									
26-31 GA	0.16	1							
32-36 GA	0.86*	0.45	1						
37-38 GA	0.83*	0.39	0.89*	1					
39-41 GA	0.86*	-0.05	0.60*	0.51*	1				
≥42 GA	0.81*	-0.00	0.70*	0.63*	0.76*	1			
Multiples									
<34 GA	0.80*	0.24	0.67*	0.64*	0.59*	0.58*	1		
34-36 GA	0.80*	-0.03	0.73*	0.62*	0.59*	0.60*	0.76*	1	
≥37 GA	0.77*	-0.10	0.70*	0.57*	0.58*	0.57*	0.71*	0.98*	1

Notes: Asterisks indicate p < 0.05. Gestational age (GA) in completed weeks (wks).

## Discussion

Our results reveal broad heterogeneity in use of cesarean delivery by week of gestation for both singleton and multiple births in Europe and the United States. Overall rates were correlated with most other sub-group rates, with the exception of very preterm births. Our analysis illustrates that there is consistency in levels of use of cesarean beyond 32 weeks GA, but there were distinct patterns in rate differences by gestational age groups across countries.

Variations in cesarean rates may result from differences in the distribution of population characteristics such as mothers’ age, parity, body mass index or country of origin, [21–23] which we were unable to take into consideration in this study. Even so, studies that have analyzed underlying differences in women’s risk profiles found that significant variations in cesarean use between countries or regions of a same country remained after risk adjustment [24–26]. Our results support the conclusions of these studies as variations in cesareans for singleton births at 40 weeks of gestation – when mortality is lowest – were at least as wide in both absolute and relative terms as that for higher-risk groups such as twins or preterm births.

Variations in delivery practices have been hypothesized to reflect population-level differences in cultural values, legal liability, specific perinatal health care characteristics and women’s preferences [14, 25, 27–29]. Countries’ structural health factors including level of subsidy for cesarean, delivery settings, and resources may also influence cesarean rates [26, 30–35]. The importance of common country-level factors is supported by the strong correlations we found between overall and sub-group rates. For instance, Nordic countries such as Finland, Sweden and Norway tended to have lower rates than other countries such as Germany, Austria, and the United States.

Among singletons, cesarean delivery rates mapped onto a general pattern which mirrors risks of adverse birth outcomes by gestational age. This pattern translates into a significant decline in cesarean rates with increasing gestational age until 40 weeks followed by a rise at 41 and 42 weeks. Additionally, in many countries, we observed an increase at 38 versus 37 weeks, most probably explained by a rise in elective cesarean delivery. In the event of a complicated pregnancy, near term extraction may be judged to confer less risk for the fetus than the benefits of a longer duration of gestation [36]. Interestingly, this rise occurred everywhere at 38 weeks and not at 37 weeks – although a birth at 37 weeks is not considered preterm – and not at 39 weeks, although many professional societies have issued recommendations advising against elective delivery before 39 weeks [37].

Our results show that reporting cesarean rates by gestational age and multiplicity provides useful additional information for countries seeking to understand and compare cesarean practices. For example, whereas cesarean rates were generally low in Sweden and Finland, very preterm rates were high. On the other hand, the United States displayed the highest overall cesarean rate but very preterm rates were lower than in many other countries. In general, there was no correlation between rates at term and at very preterm gestations, suggesting that interpretation of research on the benefits of cesarean for very preterm delivery may be independent from general attitudes towards cesarean. In fact, the benefits of cesarean delivery for very preterm babies in the absence of other obstetric indications are debated [19, 38]. Moreover, different ethical decisions related to active treatment for extremely preterm births might enhance heterogeneity between countries in obstetricians’ decisions to choose cesarean delivery [39, 40].

Similarly, there were differences in delivery practices for multiples. Thus, for instance, two countries with low overall cesarean rates, The Netherlands and Sweden, had very different rates for multiples. In addition, whereas Austria, Estonia and The Netherlands displayed similar cesarean rates for multiples throughout all weeks of gestation, Finland, Sweden and Norway had declining rates with increasing gestational age. The use of systematic cesarean sections for preterm and term twin births is also an area where scientific evidence has been largely debated [41]. In a 2013 randomized control trial, researchers showed that planned cesarean delivery did not increase or decrease the occurrence of adverse birth outcomes compared to vaginal delivery for twin pregnancies [42].

Stratified data by gestational age and multiplicity can be used to refine policies aiming to reduce unnecessary cesareans, or to evaluate uptake of national or state policies targeting certain risk groups. In the United States, for example, financial quality incentives have been implemented to discourage elective delivery before 39 weeks in hospitals and Medicaid coverage for near term elective deliveries has been phased out in states like New York, South Carolina and New Mexico [43]. Moreover, presenting cesarean rates by subgroups can be useful to assess changes in practices resulting from the publication of new studies and trials on best mode of delivery.

Our study compared cesarean delivery rates by risk groups using data from routine population-based registers in 18 countries and very few data items were missing for variables included in our analyses. The use of subgroup data from routine population registers represents a strength over other international studies based on overall cesarean rates only or institution-level data. We asked for data on gestational age in completed weeks based on the best clinical estimate, but did not have further information on how this estimate was derived. Although mode of onset and previous mode of delivery may also contribute to overall cesarean patterns, these data were unavailable. Our study covered only one year and thus our estimates are less precise in smaller European countries, especially at the extremes of the gestational age distribution.

## Conclusions

The presentation of cesarean rates by gestational age in cross-national comparisons is feasible and these data were readily available in a large number of countries. Overall cesarean rates provide a valuable summary measure of practice given the strong correlations between subgroups, with the exception of very preterm births. However, cesarean data presented by gestational age and multiplicity could make it possible to design more targeted cesarean reduction policies, and to assess changes in practice for subgroups at higher risk of cesarean delivery namely preterm, near term deliveries and multiple births.

## Abbreviations

GA: Gestational age
Wks: Weeks.
